# Poisons and Their Antidotes

**Published:** 1880-04

**Authors:** Th. Schlosser


### ARTICLE VI.

### *Poisons and their Antidotes.*

Dr. Th. Schlosser has published in the *Zeit. des Allg. Œsterreich. Apoth. Ver.* the following table of antidotes for the convenience of pharmaceutists, thus avoiding the necessity of consulting books.

*It is premised that the first thing to be done is to cause the patient to vomit: if he has already vomited there is no need to give him the emetic.*

*Aconitia.* Give an emetic composed of sulphate of copper, 15 grains, in 10 fluid drachms of water. Half to be taken at once, and if necessary after five minutes, the remainder is to be administered.

Then the following:—Tannic acid, 1 drachm. Water, 6 fl. ounces. Simple syrup, 1½ fl. ounces. Dose, a tablespoonful every five minutes.

*Ether.* Give the following in one dose:—Ammonia, 15 drops. Water, 5 fl. drachms. Then let the patient smell ammonia, apply the cold douche, and let in fresh air.

*Caustic Alkalies and their Carbonates.*—Tartaric acid, 2½ drachms. Water, 1 quart. Take a tumblerful at once, then every five minutes a teaspoonful of sweet oil and five teaspoonfuls of the tartaric acid solution.

*Caustic Lime and Lime Salts.*—Epsom salts, 5 drachms. Water, 3 ounces. Simple syrup, 1½ fl. ounces. Take at once. Then take, every fifteen minutes, two teaspoonfuls of the following: Oil of sweet almonds, 5 fl. drachms. To make an emulsion measuring three fluid ounces.

*Alcohol.* (Drunkenness.)—1. Pepsine, ½ drachm. Water, 6 ounces. Hydrochloric acid, 20 drops. A tablespoonful every five minutes, or, 2.—Ammonia, 10 drops. Water, 5 ounces. Simple syrup, ½ fl. ounce. Take at once.

*Ammonia.*—1. Concentrated acetic acid to smell, or, 2.—Vinegar, 5 fl. drachms. Water, 6 ounces. Simple syrup, ½ fl. ounce. A tablespoonful every five minutes, or, 3.—Vinegar, 1½ fl. ounces. Water, 6 ounces. Inhale hot. Cold water to wash.

*Anilin colors.*—Sulphate of copper, 15 grains. Water, 10 fl. drachms. Give one-half at once; then, if necessary, after five minutes the remainder. Then: Calcined magnesia with sufficient water to make six fluid ounces of milk of magnesia. Every half-hour a tablespoonful.

*Antimoniates and Tartar Emetic.*—Tannic acid, 45 grains. Water, 4½ fl. ounces. Simple syrup, 2 fl. ounces. A teaspoonful every five minutes.

*Arseniates.*—Milk of calcined magnesia made as above. Half a tumblerful at once, and every five minutes a tablespoonful.

*Atropia.*—1. Jaborandi leaves, 2½ drachms. Make an infusion of 6 ounces. Take one-half at once, then every half hour a tablespoonful with a tablespoonful of wine, or, 2. Muriate of pilocarpia, ¾ grain. Water, ½ fl. drachm. For subcutaneous injection.

*Baryta.*—The same as *Lead Salts.*

*Belladonna*—The same as *Atropia.*

*Bites from Dogs and Cats.*—Caustic potassa, 15 grains. Water, 1 pint. Wash out the wound, and keep linen moistened with it on the wound until the physician comes.

*Bites from Snakes.*—The same as above, or, Ammonia, 30 drops. Water, 4½ fl. ounces. Simple syrup, 1 fl. ounce. A tablespoonful every five minutes.

*Lead Salts.*—Fluid extract of senna, 1 fl. ounce. Epsom salts, 1 ounce. Warm water, ½ pint. Take in two portions at ten minutes interval.

*Bromine.*—Calcined magnesia and sufficient water to make six fluid ounces. Half a tumbler at once, then a tablespoonful every fifteen minutes.

*Brucia.*—The same as *Strychnia.*

*Cannabis Indica.*—The same as *Morphia.*

*Cantharidia.*—Sulphate of copper, 12 grains. Water, 10 fl. drachms. Half at once; the remainder, if necessary, in five minutes. Then: Powdered camphor, 45 grains. Syrup of acacia, 8 fl. ounces. Laudanum, 10 drops. Suspend the camphor well in the syrup, and add the laudanum. Dose, a tablespoonful every five or ten minutes.

*Carbolic Acid.*—Give an emetic of sulphate of copper; then milk of calcined magnesia and water, six fluid ounces. Half at once, and afterwards a tablespoonful every fifteen minutes, alternating with a tablespoonful of the oily emulsion mentioned above.

*Chloral Hydrate.*—1. Sulphate of atropia, 1–30 grain. Water, 10 fl. drachms. In two portions, with half an hour between, or, 2.—Tincture of belladonna, 10 fl. drachms. Give in the same way.

*Codeia.*—The same as *Morphia.*

*Colchicia.*—The same as *Aconitia.*

*Chloroform.*—Give ammonia to smell, then apply the cold douche and ice on the head. Then give one or two seidlitz powders, and in extreme cases the usual emetic of sulphate of copper.

*Chlorine.*—Bitter almond water, (German Pharm.) 2½ fl. drachms. Ether, 1 fl. ounce. Alcohol, 1 fl. ounce. To smell and inhale. Then: Sweet spirits of nitre, 5 fl. drachms. Syrup of acacia, 10 fl. drachms. Water, 10 fl. drachms. A tablespoonful every five or ten minutes.

*Chromic Acid and Chromates.*—Powdered iron, 75 grains. Oily emulsion, 1½ ounces. Mucilage, 1½ ounces. Shake well. One teaspoonful every five minutes, then two table-spoonfuls of water.

*Sulphuretted Hydrogen and Foul Air.*—Hoffmann's anodyne, 1 fl. ounce. Ten drops every five minutes in a teaspoonful of water. Then: Sweet spirits of nitre, 1½ fl. ounces. Pour it on a cloth and let the patient inhale it, or give chlorinated lime to smell; supply fresh air and wash the face with vinegar.

*Conia.*—Nitrate of strychnia, 1–6 grain. Water, 3 ounces. Laudanum, 30 drops. Two teaspoonfuls every fifteen minutes until one third has been used, then every half hour till one-third is left, then the same dose every hour.

*Curare.*—Nitrate of strychnia, ¾ grain. Water, 1¼ fl. drachms. For subcutaneous injection.

*Hydrocyanic Acid*—Sulphate of copper, 1–2 drachm. Water, 1 fl. ounce. Half at once, and the remainder after five minutes. Apply the cold douche.

*Digitalis.*—The same as *Morphia.*

*Poisonous Mushrooms.*—The same as *Chloral Hydrate*, or, Sulphate of atropia, 1–6 grain. Water, 1¼ fl. drachms. For subcutaneous injection.

*Hellebore.*—The same as *Aconitia.*

*Hyoscyamus.*—The same as *Morphia.*

*Insect Bites.*—Apply ammonia to the bite.

*Iodine*—Starch, 2 drachms. Water to make 5 fl. ounces. Boil, and add Milk of calcined magnesia, 5 fl. ounces. A tablespoonful every five minutes.

*Oxalic Acid and Oxalates.*—Precipitated chalk, 1 1–2 ounces. Water, 6 fl. ounces. Half at once, then a table-spoonful every ten minutes. Then, after half an hour: Fluid extract of senna, 1 fl. ounce. Sulphate of soda, 2 1–2 drachms. Water enough to make 2 fl. ounces. Take at once.

*Carbonic Acid and Carbonic Oxide.*—Give ammonia to smell, and apply the cold douche. Then: Fluid extract of ergot, 1 fl. drachm. Water, 2 fl. ounces, A teaspoonful every fifteen minutes.

*Creasote.*—Oily emulsion, half pint. Take one-quarter at once, then half a teacupful every ten minutes.

*Swallowing of a Copper Coin.*—Fluid extract of senna, 1 fl. ounce. Water, 1 fl. ounce. Sulphate of soda, 2 1–2 drachms. Take at once. This dose is for an adult. Children in proportion.

